# Capacity for conducting systematic reviews in low- and middle-income countries: a rapid appraisal

**DOI:** 10.1186/s12961-015-0012-0

**Published:** 2015-04-26

**Authors:** Sandy Oliver, Mukdarut Bangpan, Claire Stansfield, Ruth Stewart

**Affiliations:** UCL Institute of Education, 20 Bedford Way, London, WC1H 0AL UK; University of Johannesburg, House 2, Research Village, Bunting Road Campus, Auckland Park, Johannesburg, South Africa

**Keywords:** Capacity building, Capacity strengthening, Research capacity, Synthesis, Systematic review

## Abstract

**Background:**

Systematic reviews of research are increasingly recognised as important for informing decisions across policy sectors and for setting priorities for research. Although reviews draw on international research, the host institutions and countries can focus attention on their own priorities. The uneven capacity for conducting research around the world raises questions about the capacity for conducting systematic reviews.

**Methods:**

A rapid appraisal was conducted of current capacity and capacity strengthening activities for conducting systematic reviews in low- and middle-income countries (LMICs). A systems approach to analysis considered the capacity of individuals nested within the larger units of research teams, institutions that fund, support, and/or conduct systematic reviews, and systems that support systematic reviewing internationally.

**Results:**

International systematic review networks, and their support organisations, are dominated by members from high-income countries. The largest network comprising a skilled workforce and established centres is the Cochrane Collaboration. Other networks, although smaller, provide support for systematic reviews addressing questions beyond effective clinical practice which require a broader range of methods. Capacity constraints were apparent at the levels of individuals, review teams, organisations, and system wide. Constraints at each level limited the capacity at levels nested within them. Skills training for individuals had limited utility if not allied to opportunities for review teams to practice the skills. Skills development was further constrained by language barriers, lack of support from academic organisations, and the limitations of wider systems for communication and knowledge management.

All networks hosted some activities for strengthening the capacities of individuals and teams, although these were usually independent of core academic programmes and traditional career progression. Even rarer were efforts to increase demand for systematic reviews and to strengthen links between producers and potential users of systematic reviews.

**Conclusions:**

Limited capacity for conducting systematic reviews within LMICs presents a major technical and social challenge to advancing their health systems. Effective capacity in LMICs can be spread through investing effort at multiple levels simultaneously, supported by countries (predominantly high-income countries) with established skills and experience.

## Background

Since the 1980s, there has been a move towards an explicit use of evidence when making decisions about professional practice, service delivery, and public policy. This began in high-income countries (HICs) in health care and has been spreading across policy sectors and national boundaries. Systematic reviews, as scientific approaches to producing systematically and transparently quality-assessed syntheses of research, are now commonly produced in HICs and increasingly sought by clinicians, service users, professional bodies, funding agencies, and policymakers to inform their own decisions.

Efforts to increase global capacity in systematic reviewing, rather than wait for interest to spread, began with reviews of the effects of clinical interventions, with the founding of The Cochrane Collaboration in 1993 and The Joanna Briggs Institute (JBI) in 1996. The Campbell Collaboration focused efforts on education, social justice, and social welfare following an initial meeting in 1999. The Evidence for Policy and Practice Information and Coordinating Centre (EPPI-Centre) began supporting review teams in education in 2000. The Alliance for Health Policy and Systems Research (AHPSR; now hosted by the World Health Organisation (WHO)) established systematic review centres in four low- and middle-income countries (LMICs) in 2007. The same year saw the Collaboration for Environmental Evidence (CEE) being registered for charitable purposes. More recently, the International Initiative for Impact Evaluation (3ie), founded in 2008, and the UK Department for International Development (DFID) have invested in systematic reviews, and associated training and methods support, for international development (http://r4d.dfid.gov.uk/SystematicReviews.aspx).

These organisations have a shared interest in strengthening systematic review capacity in LMICs. Dimensions for strengthening capacity for health research [1] and methods for its evaluation [2] have been developed from systematic reviews of research literature and good practice. There is a complementary literature on building systems to use synthesised evidence [3]. This paper addresses the gap between the two: the capacity to synthesise research findings before making them widely available for decision-makers. It reports a rapid appraisal of LMICs current capacity and efforts to enhance capacity to synthesise the evidence available for policy decisions; evidence not only about assessing policy options, but also about understanding the nature and scale of policy problems and about policy implementation [4], as well as the applicability of findings from one setting to another.

## Methods

**Figure 1:**
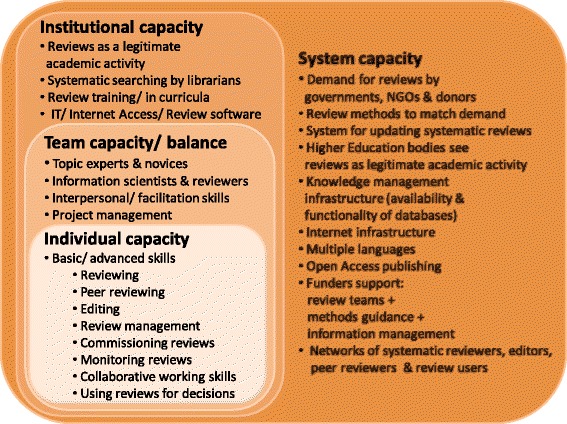
What capacity means for systematic reviewing.

Rapid appraisal was the method chosen for quickly developing an understanding of the current situation to inform future decisions about capacity strengthening. It employs a systems perspective, triangulation of data collection, and iterative data collection and analysis [5]. A system perspective was imposed with the help of a multi-level research capacity building framework [1] applied to systems for systematic reviewing (Figure 1).

Triangulation of data collection was achieved by drawing on routine management data, consulting key informants (systematic reviewers in LMICs and staff at review support organisations), and a survey of attendees at a South Asian systematic reviewing conference. Representatives of participating organisations (Cochrane and Campbell Collaborations, 3ie, EPPI-Centre) guided data collection, both initially and following discussion of initial analyses. Participating organisations were invited (April to May 2013) to contribute information and reflections on their current capacity and capacity strengthening efforts for funding, supporting, conducting, or using systematic reviews.

Information was sought by inspecting websites hosted by review support organisations (3ie, Campbell Collaboration, Cochrane Collaboration, CEE, JBI, and the EPPI-Centre) and review funders (3ie, the Australian Agency for International Development, AHPSR (WHO), and DFID); asking these organisations for relevant documents and names of people with direct experience of efforts to produce or support the production of systematic reviews in LMICs, as defined by the World Bank ^a^; broadcasting requests for information about capacity and capacity strengthening in LMICs via Twitter; inviting reviewers, managers, trainers, and funders with direct experience of producing reviews or strengthening capacity in LMICs to offer their reflections through email conversations or discussions, face-to-face or by Skype or telephone; conducting an on-line survey in May 2012 emailed to participants ^b^of a mini-Campbell Colloquium for international development held in Dhaka in December 2012; and inspecting publicly available documents about other organisations found to be relevant during the course of the study. Additional data collection, from other key informants or documents, was prompted by the emerging findings. A detailed draft report was circulated first for comment to all the participating individuals and organisations, and again to inform their own capacity strengthening activities.

This study was approved by the Research Ethics Committee of the Faculty for Childhood, Families and Health at the Institute of Education, University of London.

## Results

We present herein the challenges, useful resources, and promising activities for strengthening the capacity of individual researchers, teams, organisations, and knowledge management resources.

### Individual researchers, research teams, and networks

Networks of systematic reviewers (listed in Table 1) included LMIC researchers contributing to systematic reviews across a number of policy sectors. The largest network of a skilled workforce and established centres, both in LMICs and in HICs, is the Cochrane Collaboration, which focuses primarily on health and questions about the effects of interventions. Other networks, although much smaller, provide specialist skills in the production of reviews beyond health care and beyond questions about the effects of intervention.

**Table 1:** **Networks of systematic review centres**

**Organisations**	**Scope of systematic reviews**	**Descriptions of networks in LMICs**
Alliance for Health Policy and Systems Research (WHO) (http://www.who.int/alliance-hpsr/about/en/)	Health systems research	The Alliance funded six systematic review centres (only four currently active) in LMICs (Lebanon, South Africa, China, Chile, Uganda, and Bangladesh)
Campbell Collaboration (http://www.campbellcollaboration.org/)	Crime and justice, Education, International development, Social welfare	The International Development Review Group is based in London, UK, and is part of an Indian-based institution, and supports teams conducting international development reviews, with some of the authors being based in LMICs
Cochrane Collaboration (http://www.cochrane.org)	Health care	A network of healthcare practitioners and researchers from more than 120 countries. Cochrane has 14 Centres supporting systematic review; nearly all centres/networks have LMICs in their scope [8]
Collaboration for Environmental Evidence Centre (http://www.environmentalevidence.org/)	Environmental science	A network of researchers and managers to promote systematic reviews of environmental management; the centre has four centres including CEE Johannesburg in South Africa
EPPI-Centre (http://eppi.ioe.ac.uk/cms/)	Education and social policy, Health promotion and public health, International health systems and development, Participative research and policy	The centre in London, UK, supports teams funded to conduct systematic reviews for international development with some of the authors being based in LMICs
Joanna Briggs Institute (JBI) (http://joannabriggs.org)	Health care	JBI has 25 Centres and Evidence Synthesis groups in LMICs, many provide training to prepare systematic reviews with a focus on healthcare policy and practice

**Figure 2:**
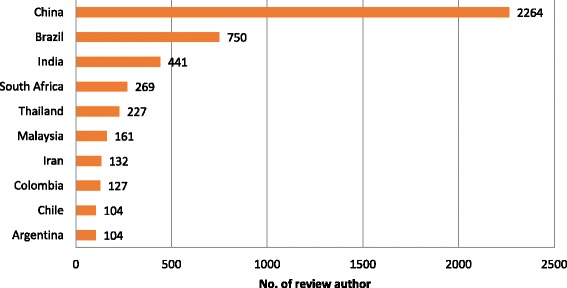
LMICs with more than 100 Cochrane review authors.

Analysis of routine management data [6] identified 60 LMICs with Cochrane review authors. Ten of these, all upper middle-income countries (MICs), currently have over 100 Cochrane review authors (Figure 2), and only 13 low-income countries (22%) have Cochrane review authors. A different ten countries, all MICs, had the most authors per capita [7] (Saint Vincent and the Grenadines, Grenada, Malaysia, South Africa, Syrian Arab Republic, Brazil, Lebanon, Thailand, Jamaica, Colombia). No LMIC has more than 10 Cochrane editors, and other specialist roles such as statisticians and information scientists or librarians are particularly scarce. The 20 LMIC institutional hosts with most Cochrane contributors (45–695) are spread across East Asia, Latin America, South Asia, Africa, and the Middle East^c^. The Cochrane Collaboration has established its own review groups and centres, often hosted by higher education institutions, to support the production of systematic reviews. Cochrane Centres, or their branches, have been established in a number of LMICs [8], including the Southern American Centre (Chile/Argentina), Andean Branch (Colombia), Central American and Spanish Caribbean Branch (Costa Rica), Caribbean Branch of US Cochrane Centre (Jamaica), and Thailand. The production of Cochrane systematic reviews in a country, irrespective of income status, is positively correlated with the presence of Cochrane centres that support reviewers within a geographical region or about a specific health scope [9].

Browsing the internet and contacting review support organisations revealed a range of approaches to developing the capacity of individuals for producing systematic reviews, including training sessions at conferences; travel stipends for training participants from LMICs; training at reviewing centres: skills-based workshops focusing on introduction to systematic reviews, protocol development, critical appraisal skills, and project management; higher education accredited face-to-face and distance learning courses; coaching programmes and workshops to instigate peer support and networking; and scholarships and stipends for author secondments to progress their review.

Strengthening the skills of individuals and review teams in LMICs often faced a language barrier, a challenge mentioned by interviewees with responsibilities for LMIC training or networking. The dominant language amongst systematic reviewing networks is English so authors from developing countries need language-focused support [10]. The magnitude of the problem varies between countries and between professional groups. For instance, in Latin America, some medics, but fewer people in other health professions, are sufficiently fluent to navigate the systematic reviewing process. Preparing the final report is less problematic for reviewers based in institutions that offer support for writing in English. Where trainers are bilingual, training novices is easier in their local language in order to produce systematic reviews that fulfil local needs. However, finding peer referees who are able to comment on reviews written in other languages is more difficult and the work may be restricted to being used locally.

### Knowledge management systems

Systematic reviews and systematic reviewers are part of a larger knowledge management system for accessing, reviewing, and sharing research; a system that holds many challenges for navigating LMIC research [11] and was raised by interviewees with responsibilities for supporting the development of search strategies for LMIC review teams. Many bibliographic databases are not well designed for systematic reviews that have geographical focus on LMICs, often about international development, for several reasons. Geographical search filters, for instance, for Africa or emerging economies [12], can be applied to key databases such as MEDLINE (Ovid) and EMBASE (Ovid), to identify studies relevant to LMICs [13], but their long string of search terms cannot be applied to all databases [14]. Many citations are poorly indexed, and not all databases have systems for importing search results into reference management or review management software. For databases that have limited search functionality, the search output can be overwhelming, and empirical research can be hidden within a wealth of other material. Some repositories do not include abstracts, making it more challenging to search for the studies and to judge the relevance of a document to a review. Much relevant literature, such as working and policy papers, is not included in databases, but only through organizational websites, contacting authors, or internet search engines. Lastly, publications in multiple languages require searching and reviewing in different languages. Despite these difficulties, guidance is available [15].

#### Access to publications

Access to research reports, while important to research generally, is essential for systematic reviewing. Access to international journals from LMIC institutions can be limited (noted as a challenge by 15% of respondents from the Campbell mini-Colloquium in Dhaka), and regional journals and national journals in LMICs are not readily accessed by HICs. These challenges, noted by researchers both in LMICs and providing distance support from a HIC, are partially overcome both by initiatives to enhance access to research (see the Global Open Access Portal [16] signposted by an experienced LMIC reviewer) and by international collaboration within review teams.

#### Review management software

Capturing relevant literature for systematic reviews requires managing large numbers of studies, which is more easily done with specialist software. However, nearly three quarters of the respondents who attended the Dhaka colloquium indicated that their organisations were not able to provide review management software. A variety of specialist review software applications support different types of reviews [17]. Those with an on-line interface ^d^are particularly valuable for supporting international teams by allowing each member of the team, and their support organisations, to access the data, so long as internet access is reliable.

#### Disseminating systematic reviews

Once systematic reviews are complete, they need to be accessed by people well placed to make use of their findings. The largest collection of systematic reviews is in *The Cochrane Library* (www.thecochranelibrary.com), to which on-line access is subsidised for LMICs [18]. Other outlets are systematic reviews packaged in special collections relevant to LMICs such as the WHO Reproductive Health Library [19].

### Working across the policy-research interface

We found three different approaches to strengthening the capacity for producing systematic reviews close to the capacity to use them: strengthening capacity to use and produce systematic reviews simultaneously; creating partnerships that span policy-research interfaces; and commissioning systematic reviews within a programme that strengthens capacity amongst the immediate review ‘customers’ as well as the review producers.

#### Strengthening capacity to use and produce systematic reviews

Systematic review support organisations typically include within their programme of work strengthening capacity the use as well as production of systematic reviews. Working with both research and policy networks helps the exchange of ideas between them. Organisations with strengthening engagement with systematic reviews in both policy and research networks as a major part of their work include the Effective Health Care Research Consortium funded by DFID [20,21], the Norwegian Satellite of the Cochrane Effective Practice and Organisation of Care Review Group [22], and 3ie [23]. The first of these is closely linked, with over 450 authors and 16 editors of the Cochrane Infectious Diseases Group, and key achievements [24] include 56 new Cochrane review authors from LMICs trained and supported to complete reviews since 2005 and helping the Global Alliance for Vaccines and Immunisation to identify, interpret, and disseminate reliable research reviews to improve the delivery of vaccines globally.

#### Strengthening networks spanning policy and research

We found ten or so networks which promote partnerships across the policy-research interface. Stakeholder partnerships between civil society, health professionals, health managers, researchers, and funders encourage research to address policy concerns and, conversely, policy or practice decisions to be informed by research. Such networks exist within and across countries [25,26] and within organisations [27]. For instance, the South Asia Cochrane Centre and Network, established in 2005 with the support of the Effective Health Care Research Consortium mentioned above, has enhanced the capacity for and the development of evidence-based health care research in Asia.

#### Strengthening review capacity ‘close to policy’

Two examples of international initiatives have strengthened capacity for systematic reviewing close to policy: the AHPSR and DFID. The AHPSR funded programme commissioned six synthesis centres to build within-country capacity for conducting reviews and liaising with policymakers. These centres encountered challenges that were raised by the nature of the research available, information and communication technology, research and knowledge resources and conventions, language barriers, and development of novice reviewers learning review skills and simultaneously facing all other challenges [28]. There was a paucity of model examples for incorporating different methodologies required for health systems research or for questions other than about effectiveness (‘what works?’), although this situation has since improved [29].

Complementary learning emerged from an evaluation of the pilot programme of systematic reviews conducted by DFID [30]. Recommendations included giving more time and guidance to the task of identifying important questions that can be appropriately addressed by systematic review methods, and for continuing engagement by policy leads with reviews in progress.

### A multi-level, complex system

So far, we have described systematic review capacity issues in terms of people (individuals and teams) and knowledge management systems. Respondents made clear that individual, team, organisational, and system capacities are all interlinked.

Although training programmes exist, participants can only make good use of them if they are closely aligned with reviews in progress. Similarly, searching skills can only be developed with access to appropriate databases and searching support provided from a distance is hampered by information scientists in one institution not having access to the same databases or platforms as review teams in another. For policy-relevant reviews, the reviewing workforce needs to be complemented by workforces with skills to commission, monitor, peer review, and edit systematic reviews for policy audiences. All of these are in short supply in LMICs.

Institutional capacity is particularly weak where systematic reviews are not yet seen as valuable as primary research and where training opportunities are limited. Developing individuals with key skills in such an environment is difficult and fails to strengthen local capacity if those skills are used only to boost careers by moving out of LMICs rather than conducting systematic reviews in and for LMICs.

Institutional capacity is only meaningful if connected to broader systems that create demand for and support the production of systematic reviews and dissemination and use of their findings. Review support organisations typically seek to encourage policy makers to draw on systematic reviews by providing guidance and training to help them do so. However, methods for systematically reviewing literature are not well developed to address all types of policy-relevant questions; more methods development and capacity strengthening is required to answer questions other than impact and to take into account the complexity of interventions and context. Some HICs provide a conducive environment where systematic reviewing is considered a legitimate academic activity comparable in status with primary research. Although higher education courses in HICs are increasingly available on-line, and therefore accessible to researchers elsewhere, little on-line training for systematic reviewing is in the form of academic accredited courses on core pathways for traditional academic careers.

## Discussion

### Summary of findings

International systematic review networks are dominated by HICs. Methods are better developed and support more readily available for systematic reviews addressing questions about effective clinical practice than for reviews addressing systems and policy questions, which are often more pressing in LMICs and require a broader range of methods.

Capacity constraints were apparent amongst individuals, review teams, organisations, and system wide. Constraints at each level limited the capacity at levels nested within it. For instance, skills training for individuals had limited utility if not allied to opportunities for review teams to practice the skills. Skills development was further constrained by language barriers, lack of support from academic organisations, and the limitations of wider systems for communication and knowledge management.

All networks host some capacity strengthening activities, although these were usually independent of core academic programmes and traditional career progression. Even rarer were efforts to increase demand for systematic reviews and to strengthen links between producers and potential users of systematic reviews.

### Strengths and limitations of the study

This rapid appraisal relied on key informants, document analysis, and a survey of conference participants linked to the organisations initiating the work. It spanned several systematic review networks with different histories which offer confirmatory evidence about the challenges to strengthening systematic reviewing capacity in LMICs. Reliable data were hard to come by and information systems designed to manage review programmes are not currently designed to readily produce reports about LMIC capacity. These systems need attention before a much more thorough situational analysis can be provided.

### Wider literature

The nascent capacity in LMICs to conduct systematic reviews reflects previous geographical analyses: LMICs are poorly served by systematic reviews about health systems, with few studies from LMICs being included, and few authors based in LMICs [31].

The combination of social and technical mechanisms required to help change happen is consistent with theories and empirical evidence about how ideas spread through organisations [32]. Help can come from people spanning organisational and cultural boundaries or knowledge brokers, preferably working in teams [33]. Lessons can be drawn from systematic reviews of research capacity strengthening more generally [1], current practice by funders [34], and other support organisations, particularly when focused on LMICs [2,35,36].

The multilevel systems approach that we took was similar to that adopted by ESSENCE (Enhancing Support for Strengthening the Effectiveness of National Capacity Efforts) on Health Research [37], an initiative between funding agencies to scale up coordination and harmonization of the research capacity investments. Their principle of donors or funders aligning with priorities of countries in which they work is supported by what is known about innovations being more likely to be taken up if they fit the “*existing values, norms, strategies, goals, skills mix, supporting technologies and ways of working*” [32].

### Systematic reviews in academia

Systematic reviews are largely conducted within universities. Sustainability within this context requires them to be at the heart of academia, part of the core business of universities which is comprised of teaching, research, and knowledge transfer. Systematic reviews are an important step in gathering research knowledge from academia and passing it to services for the public. As knowledge transfer is growing as a core activity for universities, at least in HICs, there are growing opportunities for systematic reviews to be seen as core activities too.

### The next steps

Countries with skills and experience in systematic reviewing can share these more widely but effective capacity will only be spread through investing effort at multiple levels simultaneously. The knowledge, insights, and commitment of insiders and boundary spanners is required to develop institutions that are conducive to systematic reviewing, whether they are information management organisations providing access to research or universities incorporating systematic reviews into their core business of teaching, research, and knowledge transfer.

## Endnotes

^a^LMICs were identified from the World Bank classification at http://data.worldbank.org/about/country-classifications.

^b^In total, 99 participants were contacted. Twenty two responded to the survey, including review users (n = 14), review authors (n = 11), peer reviewers (n = 7), and systematic review trainers (n = 2). One respondent held no role in relation to systematic reviewing.

^c^The five LMIC institutions with the largest number of Cochrane contributors were Sichuan University (China), Universidade Federal de São Paulo (Brazil), Christian Medical College and Hospital (India), University of Cape Town (South Africa), and First Affiliated Hospital of Guangxi Medical University (China).

^d^Examples of software and the web pages are: Revman (http://tech.cochrane.org/Revman), EPPI-Reviewer (http://eppi.ioe.ac.uk/cms/Default.aspx?alias=eppi.ioe.ac.uk/cms/er4), DistillerSR (http://distillercer.com/products/distillersr-systematic-review-software/), and Sumari (http://joannabriggs.org/sumari.html).

## Abbreviations

3ie: International Initiative for Impact Evaluation
AHPSR: Alliance for Health Policy and Systems Research
CEE: Collaboration for Environmental Evidence
DFID: UK Department for International Development
EPPI-Centre: Evidence for Policy and Practice Information and Coordinating Centre
HICs: High-income countries
JBI: Joanna Briggs Institute
LMICs: Low- and middle-income countries
WHO: World Health Organisation

## References

[1] Cooke J (2005). A framework to evaluate research capacity building in health care. BMC Fam Pract..

[2] Bates I, Akoto AY, Ansong D, Karikari P, Bedu-Addo G, Critchley J (2006). Evaluating health research capacity building: an evidence-based tool. PLoS Med.

[3] Oxman AD, Fretheim A, Schunemann HJ, SURE (2006). Improving the use of research evidence in guideline development: introduction. Health Res Policy Syst.

[4] Lavis JN (2009). How can we support the use of systematic reviews in policymaking?. PLoS Med.

[5] Beebe J (1995). Basic concepts and techniques of rapid appraisal. Hum Organ.

[6] The Cochrane Collaboration’s information management system for managing documents and contacts details (Archie). http://tech.cochrane.org/archie.

[7] Population figures estimated for 2010 from World Population Prospects: The 2012 revision. http://esa.un.org/wpp/. Accessed 6 June 2013.

[8] Cochrane community. http://community.cochrane.org/contact/centres.

[9] Gøtzsche P, Tendal B, Clarke M (2011). Review production in The Cochrane Collaboration – where is it happening and why? Cochrane Methods Cochrane Methods Cochrane. DB Syst Rev..

[10] Gillies D, Maxwell H, New K, Pennick V, van der Wouden H, Fedorowicz Z, et al. The Cochrane Collaboration: a collaboration-wide survey of Cochrane authors; 2009. http://community.cochrane.org/collaboration-wide-survey-of-cochrane-authors.

[11] Stansfield C, Weightman AL, Kavanagh J, Johansen M (2013). Cochrane update: identifying health-related research resources relevant to low- and middle-income countries. J Public Health (Oxf).

[12] ISSG Search Filters Resource. https://sites.google.com/a/york.ac.uk/issg-search-filters-resource/home. Accessed June 2013.

[13] Norwegian Satellite of the Cochrane Effective Practice and Organisation of Care Group, LMIC filters. http://epocoslo.cochrane.org/lmic-filters. Accessed June 2013.

[14] Pienaar E, Grobler L, Busgeeth K, Eisinga A, Siegfried N (2011). Developing a geographic search filter to identify randomised controlled trials in Africa: finding the optimal balance between sensitivity and precision. Health Info Libr J.

[15] Hammerstrøm K, Wade A, AMK Jørgensen. Searching for studies: a guide to information retrieval for Campbell systematic reviews. The Campbell Library. 2010.http://www.campbellcollaboration.org/lib/project/179/. 10.1002/cl2.1433 PMC11386270 39258215

[16] Global Open Access Portal. http://www.unesco.org/new/en/communication-and-information/portals-and-platforms/goap/.

[17] Brunton J, Thomas J, Gough D, Oliver S, Thomas J (2012). Information management in reviews. Introduction to systematic reviews.

[18] Overview of access options for the Cochrane Library. http://community.cochrane.org/editorial-and-publishing-policy-resource/overview-access-options-cochrane-library.

[19] The WHO Reproductive Health Library. http://apps.who.int/rhl/en/.

[20] Effective Health Care Research Consortium. http://www.evidence4health.org/.

[21] Young T, Garner P, Kredo T, Mbuagbaw L, Tharyan P, Volmink J (2013). Cochrane and capacity building in low- and middle-income countries: where are we at?. Cochrane Database Syst Rev..

[22] Norwegian Satellite of the Cochrane Effective Practice and Organisation of Care Group, Scope of our work. http://epocoslo.cochrane.org/scope-our-work.

[23] Policy Influencing Activities. http://www.3ieimpact.org/en/about/what-3ie-does/policy-influenc/.

[24] Evidence for Health. Key achievements. http://www.evidence4health.org/about-ehcrc/key-achievements/.

[25] Regional East African Community Health (REACH) Policy Initiative Project 2015. http://www.eac.int/health/index.php?option=com_content&id=96&Itemid=125. Accessed 2013.

[26] WHO. EVIPNet for better decision making. Geneva: World Health Organisation; 2012.

[27] McDonald S, Turner T, Chamberlain C, Lumbiganon P, Thinkhamrop J, Festin MR (2010). Building capacity for evidence generation, synthesis and implementation to improve the care of mothers and babies in South East Asia: methods and design of the SEA-ORCHID Project using a logical framework approach. BMC Med Res Methodol..

[28] Florenzano F, Benitez J, Pantoja T (2010). Identifying methodological challenges in conducting systematic reviews of health policy and systems research: a study case in low- and middle-income countries.

[29] Gough D, Thomas J, Oliver S (2012). Clarifying differences between review designs and methods. Syst Rev..

[30] Rose P, Battock M. Review of the DFID systematic review programme. 2012. http://r4d.dfid.gov.uk/Output/193302/Default.aspx. Accessed 29 July 2013.

[31] Wilson MG, Moat KA, Lavis JN (2013). The global stock of research evidence relevant to health systems policymaking. Health Res Policy Syst..

[32] Greenhalgh T, Robert G, Macfarlane F, Bate P, Kyriakidou O (2004). Diffusion of innovations in service organizations: systematic review and recommendations. Milbank Q.

[33] Long JC, Cunningham FC, Braithwaite J (2013). Bridges, brokers and boundary spanners in collaborative networks: a systematic review. BMC Health Serv Res..

[34] Boyd A, Cole DC, Cho DB, Aslanyan G, Bates I (2013). Frameworks for evaluating health research capacity strengthening: a qualitative study. Health Res Policy Syst..

[35] Cole D, Kakuma R, Fonn S, Izugbara C, Thorogood M, Bates I (2012). Evaluations of health research capacity development: a review of the evidence. Am J Trop Med Hyg.

[36] Posthumus H, Martin A, Chancellor T. A systematic review on the impacts of capacity strengthening of agricultural research systems for development and the conditions of success. London: EPPI-Centre, Social Science Research Unit, Institute of Education, University of London

[37] The ESSENCE on Health Research Initiative. Planning, monitoring and evaluation framework for capacity strengthening in health research - ESSENCE Good Practice. 2014. http://whqlibdoc.who.int/hq/2011/TDR_essence_11.1_eng.pdf?ua=1. Accessed 9 December 2014.

